# Cold-raising unmasks sleep disruption in a *Drosophila* Alzheimer’s disease model

**DOI:** 10.17912/micropub.biology.001689

**Published:** 2025-06-19

**Authors:** Ananya Nair, Allison J. Yearwood, Brandi N. Besednjak, Mikayla M. Cully, Reece Turner, Joseph L. Bedont

**Affiliations:** 1 Kent State University, Kent, Ohio, United States

## Abstract

Chronic sleep loss is a risk factor for Alzheimer's disease (AD), and reduced and fragmented sleep is increasingly appreciated as an early-onset diagnostic and potential therapeutic target for AD. However, robustly modeling AD-like sleep deficits in fruit flies has often been challenging. We report that cold-raising unmasks deficits in sleep duration, fragmentation, and latency in one such model pan-neuronally expressing a highly pathogenic AD-associated amyloid species. This sensitized model provides a promising platform for identifying potential metabolic, proteostatic, glymphatic, and other candidate mediators bidirectionally linking sleep and AD.

**Figure 1. Sleep deficits in flies warm- or cold-raised with pan-neuronal expression of pathogenic amyloid or control proteins.**
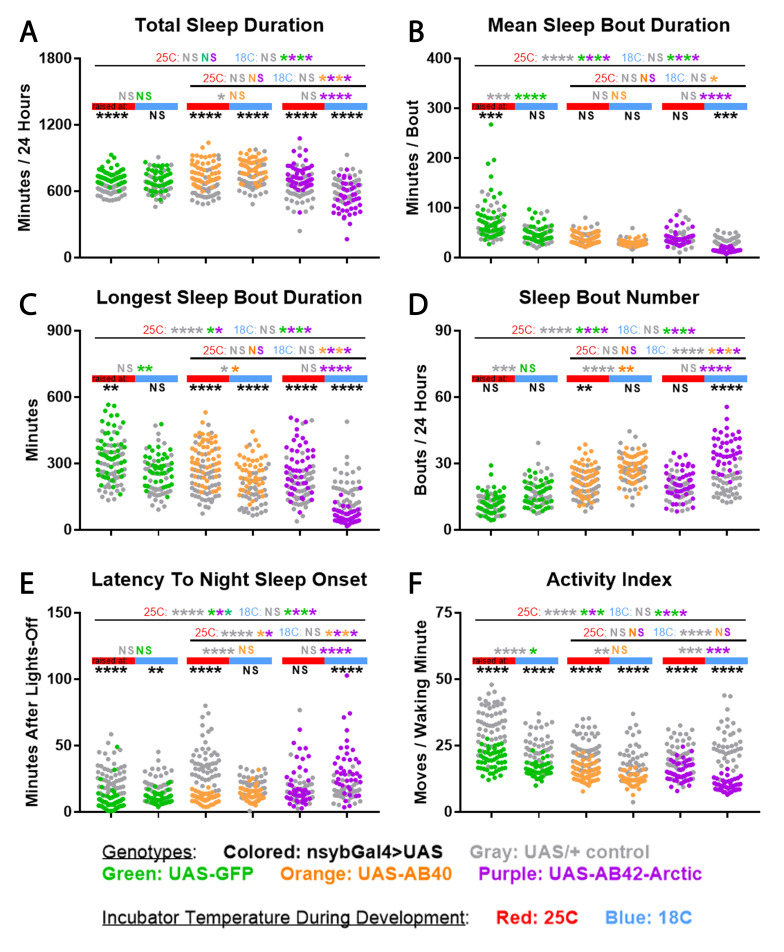
Panels show 24-hour sleep duration **(A)** ; sleep consolidation metrics ( **B:** mean sleep bout duration, **C:** longest sleep bout duration, **D:** number of sleep bouts); latency to sleep onset after ZT12 lights off **(E)** ; and activity index, defined as movements detected per waking minute **(F)** . All data was collected from n=39-50 mated adult male flies per group. Data from individual flies (one animal / dot) with nsyb-Gal4 driven over-expression of benign control protein GFP (green), less pathogenic control amyloid AB40 (orange), or highly pathogenic experimental amyloid AB42-Arctic (purple) are shown overlaid on their respective UAS genetic controls (gray). Separate pools of flies were raised at either 25C (in-line with red bar) or 18C (in-line with blue bar), but all flies were at 25C throughout sleep measurement. All data was analyzed by two-way ANOVA with post-hoc Tukey tests. Comparisons shown are within-column Gal4>UAS vs UAS-control (bottom row black asterisks) ; within-genotype 25C-raised vs 18C-raised Gal4>UAS [colored asterisks] or UAS-control [gray asterisks] (second from bottom row, directly over bars indicating developmental temperature) ; and between-genotype comparisons of control protein vs pathogenic AB42-Arctic Gal4>UAS [colored asterisks] or UAS-control [gray asterisks] at the indicated developmental temperature (top two rows). *p<0.05, **p<0.01, ***p<0.001, ****p<0.0001, NS=not significant.

## Description

Alzheimer's disease (AD) presents a looming health crisis, expected to afflict ~130 million people worldwide with dementia by 2050 ( *World Alzheimer's Report* , 2015). AD pathology progresses over decades, with defective clearance and accumulation of toxic beta-amyloid in the brain preceding cognitive decline by years (Bateman et al., 2012; Jack et al., 2013). Markers and interventions during this latency period offer opportunities to head off this costly and debilitating disease.

Sleep interacts bidirectionally with AD pathology from early on in the disease course, giving it potential on both fronts (Lucey, 2020). Cognitively normal older adults reporting sleep disturbance often exhibit brain amyloid accumulation, suggesting sleep disruption is an early biomarker of AD (Lucey, 2020). Sleep also regulates critical cellular processes including proteostasis, glymphatics, and metabolism (Bedont et al., 2023, 2021; Haynes et al., 2024; Shekhar et al., 2023; Tudor et al., 2016; Xie et al., 2013). These pathways are coupled to AD pathology (Ghosh et al., 2020; Ju et al., 2022; Liu et al., 2017; Xie et al., 2020), and may mediate sleep's role in controlling amyloid accumulation (Lucey, 2020).

The fruit fly *Drosophila melanogaster's* powerful genetics and amenability to behavioral screening have made it an invaluable model for studying cellular pathways that have separately been linked to AD and sleep (Allada et al., 2017; Feeney et al., 2025; Prüßing et al., 2013). However, sleep loss reported in amyloid over-expressing fruit flies has often been of modest magnitude (Belfer et al., 2021; Gerstner et al., 2017; Song et al., 2017; Tabuchi et al., 2015) or undetected entirely (Perlegos et al., 2024). Amyloid-driven sleep fragmentation reflecting reduced sleep quality, while sometimes more robust, has also not always been consistent. This complicates mechanistic studies in the fly interrogating the actors that bidirectionally couple sleep to AD pathology.

We report here a serendipitous observation that cold-raising at 18C instead of 25C markedly unmasks amyloid-driven sleep loss and fragmentation in adult male flies whose behavior was recorded at 25C. We utilized the Gal4 transcription factor / UAS cis element expression system to broadly express a highly pathogenic AB42 amyloid bearing the toxicity-enhancing Arctic point mutation, relatively benign AB40 control amyloid, or the fully benign control protein GFP in neurons using *nsyb-Gal4* (Brand and Perrimon, 1993; Crowther et al., 2005; Nilsberth et al., 2001; Pauli et al., 2008). UAS-only controls for each over-expression construct were also included. Several sleep and activity metrics were compared for all of these flies:

-- **Total Sleep Duration** : *nsyb>AB42-Arctic* was the only genotype with reduced total sleep time after 18C-raising compared to 25C-raising, driving significantly decreased total sleep compared to all 3 appropriate genetic controls ( *UAS-AB42-Arctic* , *nsyb>AB40* , and *nsyb>GFP* ) in 18C-raised but not 25C-raised flies ( Figure 1A ).

-- **Sleep Fragmentation (3 Metrics)** : While control genotypes showed modest but significant and/or insignificant trends toward fragmentation of sleep (shorter mean bouts, shorter longest bouts, and increased bout number) when raised at 18C compared to 25C, these developmental temperature effects were uniformly much stronger in *nsyb>AB42-Arctic* flies ( Figure 1B- D). This drove significantly shortened mean and longest sleep bout duration, and increased sleep bout number, in *nsyb>AB42-Arctic* compared to all 3 appropriate genetic controls in 18C-raised but not 25C-raised flies ( Figure 1B- D).

-- **Latency to Night Sleep** : Sleep initiation after nightfall was delayed by 18C-raising only in *nsyb>AB42-Arctic* flies ( Figure 1E ). This drove increased *nsyb>AB42-Arctic* sleep latency after nightfall compared to all 3 appropriate genetic controls in 18C-raised but not 25C-raised flies ( Figure 1E ).

-- **Activity Index** : Finally, most genotypes were significantly more sluggish or trended similarly after 18C-raising, with the exception of *UAS-AB42-Arctic* control. Activity reduction by 18C-raising may be somewhat more pronounced in *nsyb>AB42-Arctic* , but these flies had significantly reduced activity index compared to 2/3 appropriate genetic controls after both 18C- and 25C-raising. Since hyperactivity but not hypoactivity could confound *nsyb>AB42-Arctic* sleep loss, fragmentation, and delay, activity confounds do not explain away the unmasking of *nsyb>AB42-Arctic* sleep defects after 18C-raising.

We do not directly interrogate *why* cold-raising unmasks amyloidogenic sleep defects in our model, but the literature implicates several factors. Studies with wild-type flies generally find that cold-raising is associated with hypoactivity in adulthood, albeit in a manner influenced by strain (Cavieres et al., 2016; Crill et al., 1996; Gibert et al., 2001; Klepsatel and Gáliková, 2022). This is consistent with the reduced activity index we observe in most genotypes raised at 18C compared to 25C ( Figure 1F ) and may explain much of the variance in adult activity across genotypes driven by developmental temperature. More recently, the first work was published assessing developmental temperature effects on adult fly sleep. Cold-raised Indian but not Slovakian fly strains appeared to have modestly decreased and fragmented adult sleep at 25C, compared to flies raised at 25C (Yadav et al., 2025). We observed a similar effect for fragmentation, but not total sleep, in our control groups ( Figure 1A- D). Importantly, known main effects of developmental temperature on sleep do not obviously predict *nsyb>AB42-Arctic* specific sleep defects unmasked by cold-raising. That said, we cannot rule out an interaction of amyloid pathology with structural changes driven by developmental temperature, such as thermally plastic wiring of the brain's circuitry (Peng et al., 2007; Sigrist et al., 2003), as a possible contributor to *nsyb>AB42-Arctic* sleep phenotypes.

Reduced developmental expression of *AB42-Arctic* at 18C vs 25C likely contributes to the unmasking we observe. Cold temperatures reduce Gal4 transcription factor activity, and amyloid pathology is dose dependent (Duffy, 2002; Meyer-Luehmann et al., 2003). Consistent with this, continuous maintenance of a Gal4> APP ,BACE fly AD model at 18C lowers amyloid dose, and in turn delays the onset of amyloid aggregation, abnormal brain morphology, and memory deficits (Mhatre et al., 2014). That said, most prior studies of amyloid-driven sleep deficits in *Drosophila* included experiments that restricted Gal4 expression during development. Most used Geneswitch Gal4 drivers requiring mifepristone exposure to minimize amyloid expression during development (Gerstner et al., 2017; Perlegos et al., 2024; Tabuchi et al., 2015), although use of a temperature-sensitive Gal80 repressor of Gal4 activity for this purpose has also been reported (Song et al., 2017). Thus, reduced developmental amyloid expression at 18C is likely only a partial explanation for the unmasking of *nsyb>AB42-Arctic* sleep phenotypes with cold-raising. A higher ceiling on expression from an uninhibited Gal4 in adulthood, compared to Geneswitch and/or Gal80-gated expression even under permissive conditions, may also contribute.

Finally, incubator temperature directly sets the temperature experienced by amyloid proteins in exothermic species like the fruit fly. Ambient temperature history has previously been shown to modify the structure and pathogenicity of toxic amyloids (Gursky and Aleshkov, 2000; Kusumoto et al., 1998; Özcan et al., 2020), and to modulate pathology and cognitive deficits even in an endothermic mouse model of AD (Jung et al., 2022). One likely contributor to the unmasking of sleep deficits we observe is that cold-raising may disproportionately favor the formation of shorter amyloid oligomers most likely to inhibit sleep, over longer oligomers that can actually be sleep promoting (Özcan et al., 2020).

In conclusion, while the precise mechanisms remain unclear, we demonstrate that cold-raising markedly unmasks the modeling of amyloid-driven sleep loss, fragmentation, and delayed initiation at nightfall in the *nsyb>AB42-Arctic* fruit fly model of AD. We hypothesize that these effects of cold-raising may generalize to other amyloid-based fly models of AD, offering a cost-effective, convenient, and fast means of improving the modeling of amyloid-driven sleep disturbance in the fruit fly. This offers particular potential as a means of sensitizing *Drosophila* AD models for studies of the metabolic and proteostatic mechanisms that likely link amyloid pathology and sleep symptoms. Cold-raising may also offer an avenue for unmasking sleep deficits in *Drosophila* models of other neurodegenerative diseases, many of which have also been reported to exhibit inconsistent sleep phenotypes (Perlegos et al., 2024).

## Methods

Flies and Husbandry

All alleles were back-crossed to iso31 at least 5 times, and maintained as separate stocks crossed for these experiments. All flies were raised in Percival or Darwin incubators on Janelia (J) diet provided by LabExpress under a 12hour:12hour light:dark cycle at temperatures of either 18C or 25C and ~65% humidity. Excluding virgins by morphology, mated male flies were quickly collected at room temperature (generally within a short ~15-30 minute window to minimize thermal disturbance during collection) and returned to their raising temperature until being loaded for the sleep experiment, generally the next day.

Sleep Experiment

~1-5 days post-eclosion mated male flies were anesthetized with carbon dioxide and loaded into locomotor tubes with 5% sucrose / 2% agar food, placed in DAM5H monitors (Trikinetics), and moved to a behavior incubator at 25C temperature and unchanged light cycle + humidity for the duration of the experiment. Sleep was then measured from the moves (MT) metric for 5 full days of recording (excluding “Day0” loading day). To minimize artifacts from anesthesia and adaptation to the locomotor tubes, the 2 ^nd^ -5 ^th^ full days of recording were analyzed using DAMfilescan and a custom Matlab pipeline to compute all sleep metrics and averaged, as published previously (Bedont et al., 2023; Hsu et al., 2021).

Statistics

2-way ANOVA model + Tukey host-hoc hypothesis testing was run for all sleep and activity metrics in JMP 13. All graphs were generated in GraphPad Prism 7.

## Reagents

**Table:** 

**Referenced Allele**	**Full Genotype and References**	**Obtained From:**
nsyb-Gal4	;;nsyb-Gal4 (5x iso31) (Pauli et al., 2008)	Lab of Amita Sehgal
UAS-GFP	;;UAS-mCD8::GFP.L (5x iso31)	Bloomington #5130
UAS-AB40	;;UAS-AB40 (5x iso31) (Iijima et al., 2004)	Lab of Mark Wu
UAS-AB42-Arctic	;;UAS-AB42-Arctic (5x iso31) aka AlzArc2: (Crowther et al., 2005)	Lab of Mark Wu
